# Effects of C Addition on the Microstructures of As-Cast Cu–Fe–P Alloys

**DOI:** 10.3390/ma12172772

**Published:** 2019-08-28

**Authors:** Wei Chen, Xiaona Hu, Wei Guo, Jin Zou, Keming Liu, Deping Lu, Dunqiang Tan

**Affiliations:** 1 Institute of Applied Physics, Jiangxi Academy of Sciences, Nanchang 330096, China; 2 School of Materials Science and Engineering, Nanchang University, Nanchang 330031, China; 3 Jiangxi Key Laboratory for Precision Drive and Control, Nanchang Institute of Technology, Nanchang 330099, China

**Keywords:** Cu–Fe–P alloy, carbon addition, microstructure, second phase particles

## Abstract

Effects of C addition on the microstructures of as-cast Cu–Fe–P (mass fraction) alloys were systematically investigated. The results show that C addition can refine the matrix microstructure and make Fe particles finer. The Fe particles observed in both the non-C-alloyed and C-alloyed specimens are α-Fe particles, which possess a body-centered cubic (bcc) structure with a Nishiyama–Wassermann orientation relationship with the matrix. C is reported to be an γ-Fe stabilizer in the literature. The reason for the difference between the phases of Fe particles observed in this study, and that reported in the literature, are finally discussed. Additionally, C addition facilitates the decomposition of the supersaturated solid solution which occurs by the simultaneous precipitation of very fine Fe particles. Such initial decomposition product has an face-centered cubic (fcc) structure with a cube-on-cube orientation relationship with the matrix.

## 1. Introduction

The Cu–Fe system alloys are excellent candidates for industrial applications due to their low cost and good mechanical and physical properties [1,2]. However, these alloys normally exhibit a lower electrical conductivity and microhardness compared with that of other Cu-based alloys, which is attributed to the relatively higher solubility of Fe in Cu at high temperatures and slow kinetics of Fe precipitation at lower temperatures [3]. In order to improve the strength and conductivity of the Cu–Fe system alloys, many methods including thermo-mechanical treatments and adding other alloying elements have been widely investigated [4,5,6,7,8,9,10,11]. It is found that each element added into the Cu–Fe system alloys can influence the mechanical and electrical properties in various distinct ways. For example, P stabilizes the cold worked microstructures in the Cu–Fe alloys [7], B and Ce increase the recrystallization temperatures of the Cu–Fe–P alloys [4], and Ag decreases the Fe solubility and accelerates the Fe precipitation kinetics in the Cu–Fe alloys [5].

C is seldom used as the alloying element for Cu-based alloys due to its virtually zero solubility in the Cu matrix [12]. Recently, Jeong et al. [13] and Kim et al. [14] found that C addition and thermo-mechanical treatments can improve the mechanical properties of the Cu–Fe–P alloys by facilitating the finer distribution of Fe particles. Guo et al. [15,16] reported that C addition and cold rolling can significantly improve the mechanical properties of the Cu–Fe alloys by introducing the martensitic transformation (γ-Fe to α-Fe) of nano-sized Fe–C particles. However, in order to fully understand the influences of C on the Cu–Fe system alloys, it is necessary to accurately characterize the initial as-cast microstructures of the non-C-alloyed and C-alloyed samples, because the strengthening particles are usually affected in size or phase by various processes.

It is well known that C addition can induce liquid phase separation in the Cu–Fe alloys with relative high contents of Fe (commonly larger than 5 wt.%) [17,18]. Therefore, this study was focused on the effects of C on the Cu–Fe alloys with relatively low contents of Fe (smaller than 5 wt.%), i.e., a nominal composition of Cu–1.8Fe–0.02P. The macroscopic and microscopic structures, second phase particles, and initial decomposition product of the non-C-alloyed and C-alloyed specimens were analyzed in detail.

## 2. Experimental Section

Cu–1.8Fe–0.02P (wt.%) alloys with an addition of 0 wt.%, 0.01 wt.%, 0.05 wt.% and 0.1 wt.% C, which are referred to as C00, C01, C05 and C10 respectively, were produced by melting electrolytic copper (99.99 wt.%), industrial pure iron (99.97 wt.%), and intermediate Fe–5C alloy in a magnesia crucible in the vacuum medium-frequency induction furnace, and casting into a graphite mold with an inner diameter of 36 mm. The contents of C were determined by a CS-2800 type carbon-sulfur analyzer (Central Iron & Steel Research Institute, Beijing, China), and other alloying elements were determined by a SPECTROMAXx type spark emission spectroscope (Spectro, Kleve, Germany). The measured compositions of the as-cast alloys are shown in Table 1. It is found that the C-alloyed specimens (C01, C05 and C10) have nearly the same C contents (0.011–0.015 wt.%) although different amounts of C (0.01–0.10 wt.%) were added into the alloy melts. This is because the solubility of C in the Cu–1.8Fe–0.02P alloy is rather limited [14]. Some of the C added into the C05 and C10 specimens that was not detected by the carbon-sulfur analyzer should exist in the form of graphite, which floated up to the top of the samples together with other slag during the casting process, according to the analysis of the equilibrium phase diagrams of the Cu–Fe–C ternary system [18].

The crystal structures of the second phases and matrix were identified by X-ray diffraction (XRD, D/MAX-1200, Tokyo, Japan). Macroscopic and microscopic structures of the as-cast alloys were observed by an optical camera and a FEI NanoSEM Nova 430 field emission scanning electron microscope (SEM, Lausanne, Switzerland) equipped with an energy dispersive spectrometer (EDS), respectively. Samples for optical and SEM observation were etched using a solution of 10 g FeCl_3_, 25 mL HCl and 100 mL H_2_O. Characterizations of the Fe particles and initial decomposition product were carried out on a Talos F200x transmission electron microscope (TEM, Hillsboro, OR, USA) operated at a voltage of 200 kV. The size and element distribution of the Fe particles and initial decomposition product were determined using scanning transmission electron microscopy high-angle annular dark-field (STEM-HAADF) images. Thin foils for TEM observation were first mechanically ground to a thickness of 30 μm and then twin-jet polished in a solution of 30% HNO_3_ and 70% CH_3_OH at a temperature of −25 °C.

## 3. Results

### 3.1. Macroscopic and Microscopic Structures

**Figure 1:**
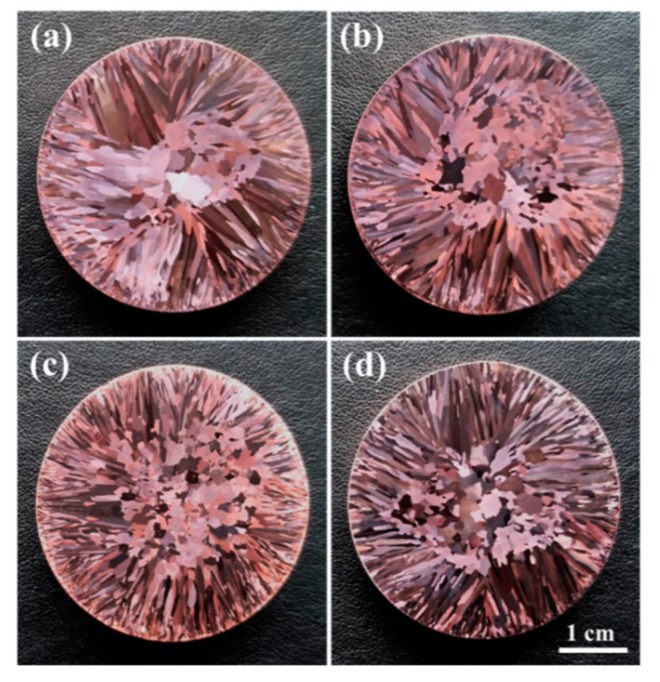
Cross-sectional macrostructures of (**a**) C00, (**b**) C01, (**c**) C05 and (**d**) C10 specimens, respectively.

Differences in the macrostructures of the non-C-alloyed and C-alloyed specimens were examined. As shown in Figure 1a, the macrostructure of the non-C-alloyed specimen is composed of coarse columnar and equiaxed grains. With the addition of C, the columnar-to-equiaxed transition and decrease of grain size are observed (Figure 1b–d). This result indicates that C addition can refine the grains of the Cu–1.8Fe–0.02P alloys. It is noted that the C05 specimen has the finest grains (Figure 1c). By considering the fact that the C05 specimen has a much higher P content than other C-alloyed specimens (Table 1), it indicates that the increase of P content also has a certain refining effect on the grains of the alloys under investigation.

**Figure 2:**
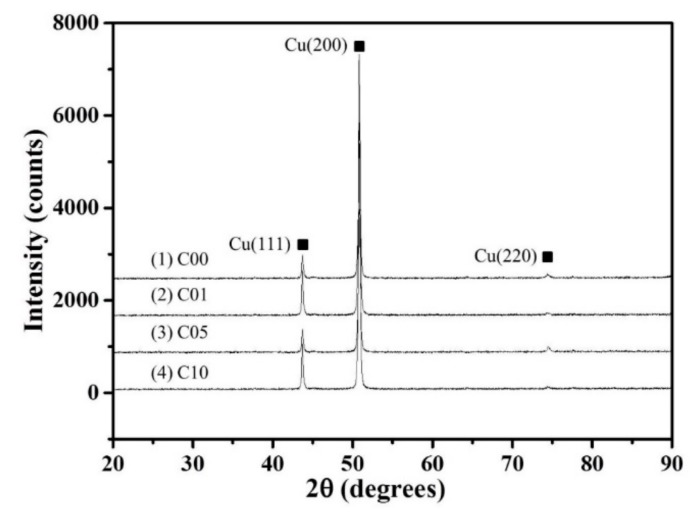
XRD patterns of Cu–1.8Fe alloys with and without addition of C.

**Figure 3:**
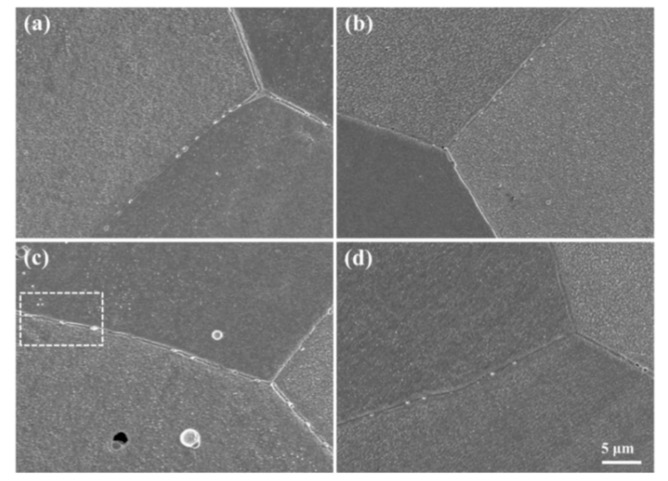
Typical SEM images of (**a**) C00, (**b**) C01, (**c**) C05 and (**d**) C10 specimens, respectively.

**Figure 4:**
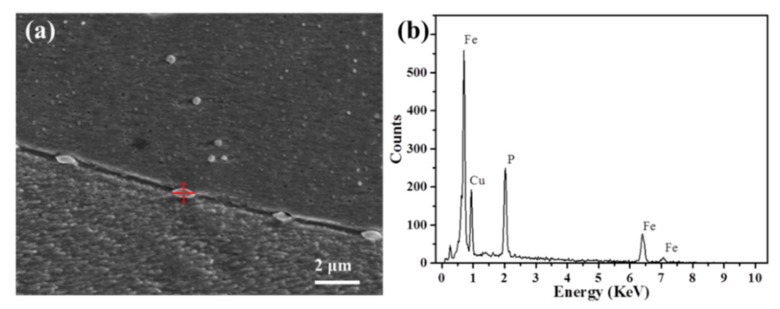
(**a**) An enlarged SEM image of the enclosed area with dotted lines in Figure 3c and (**b**) energy dispersive spectrometer (EDS) analysis of particles in (**a**).

The XRD patterns of the Cu–1.8Fe alloys with and without the addition of C are shown in Figure 2, where obvious fcc ε-Cu phase can be observed. No diffraction peaks of iron, carbon or other second phases can be observed, which is attributed to the limited alloying elements contents as measured in Table 1. Figure 3 shows the typical SEM images of the non-C-alloyed and C-alloyed specimens. A uniform distribution of fine second phase particles is observed in the C00 (Figure 3a), C01 (Figure 3b) and C10 (Figure 3d) specimens, while bimodal distribution of coarse and fine particles is observed in the C05 (Figure 3c) specimen. Closer examination indicates that the particles located inside the grains are nearly spherical in shape, while those located on the grain boundaries are roughly elliptical in shape (Figure 4a). EDS analyses on the particles show that they are comprised of Fe, Cu and P elements with an atom ration of 3:1 for Fe and P (Figure 4b). Therefore, these particles are identified as Fe_3_P intermetallic particles with the size ranging from 150 nm to 600 nm. There is no significant difference in the morphology and size of the Fe_3_P particles in the specimens of C00, C01 and C10, indicating that the addition of C has no obvious effect on the Fe_3_P particles. The larger size and higher density of Fe_3_P particles in the C05 specimen compared to other specimens is attributed to its higher P content.

**Figure 5:**
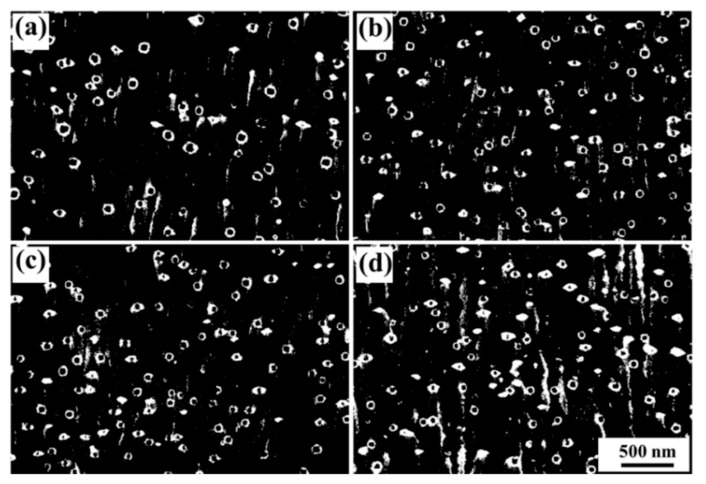
High-magnification SEM images of (**a**) C00, (**b**) C01, (**c**) C05 and (**d**) C10 specimens, respectively.

Smaller second phase particles with the size ranging from 52 nm to 86 nm are observed in the high-magnification SEM images of the non-C-alloyed and C-alloyed specimens (Figure 5). For easy identification of second phase particles, the contrast of the SEM images was adjusted. These particles are supposed to be Fe particles which are later confirmed in the TEM study. The addition of C appears to reduce the size and increase the number density of the Fe particles. However, more details need to be investigated under TEM observation.

### 3.2. Fe Particles and Initial Decomposition Product

**Figure 6:**
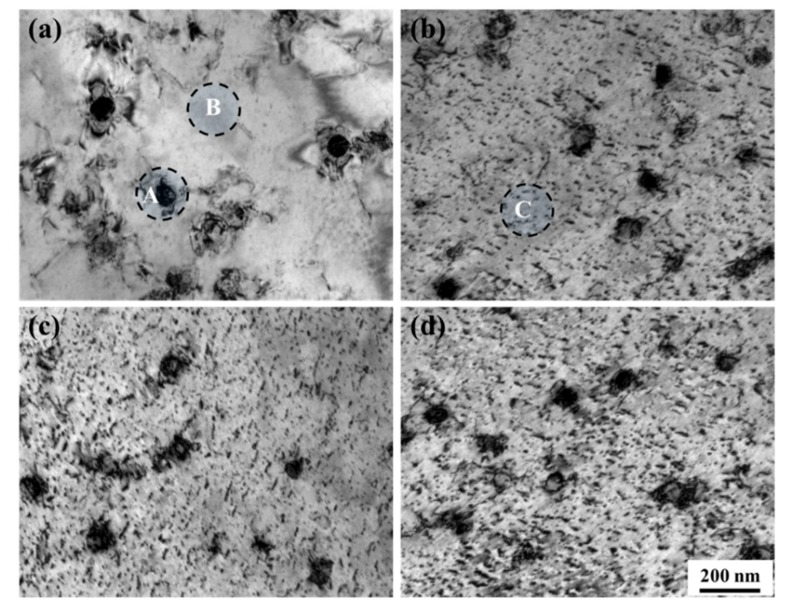
Bright-field TEM images of (**a**) C00, (**b**) C01, (**c**) C05 and (**d**) C10 specimens, respectively.

**Figure 7:**
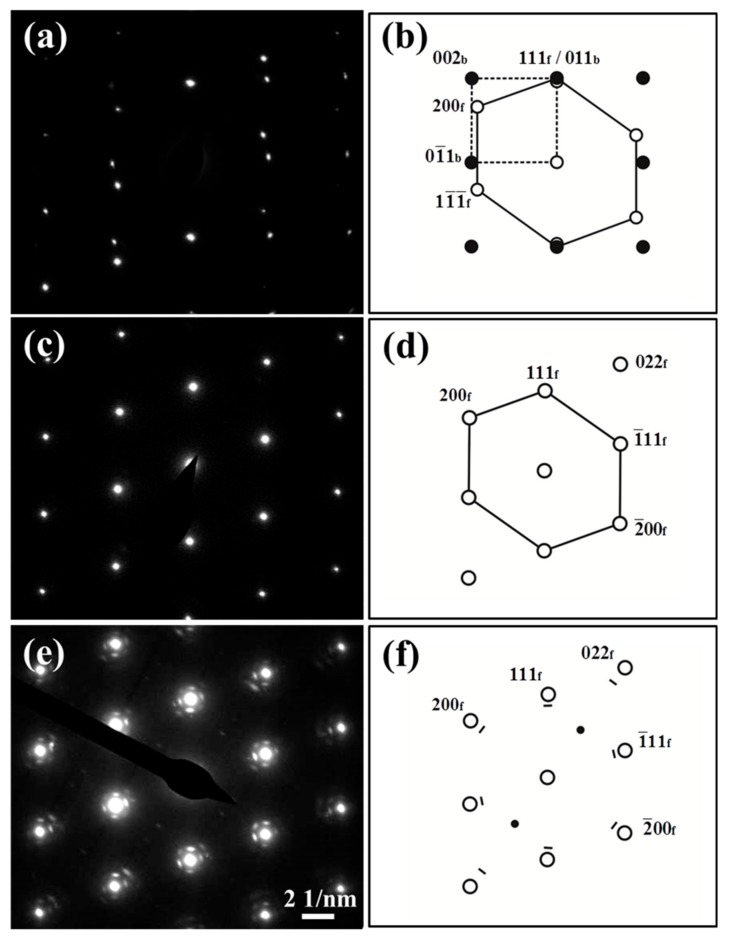
(**a**,**c**,**e**) [$01\bar{1}$]_f_ SAD patterns taken from the areas of A, B and C indicated in Figure 6a,b, respectively and (**b**,**d**,**f**) schematic diffraction patterns corresponding to (**a**), (**c**) and (**e**), respectively. (**b**), (**d**) and (**f**) show positions of the reflections (⚫) from the bcc α-Fe particle, reflections (⚪) from the fcc Cu matrix, fundamental (▬) and superlattice (●) reflections from the initial decomposition product. All other reflections in (**e**) arise due to double diffraction.

Figure 6 shows bright-field TEM images of the non-C-alloyed and C-alloyed specimens. Figure 7 shows the selected area diffraction (SAD) patterns and corresponding keys taken from the regions of A and B in Figure 6a and C in Figure 6b. SAD analysis indicates the particles (labeled A) appearing in the non-C-alloyed specimen are α-Fe particles (Figure 6a), which have a bcc structure with a Nishiyama–Wassermann (N–W) orientation relationship (OR) with the Cu matrix, i.e., (111)_f_//(011)_b_, [$01\bar{1}$]_f_//[100]_b_ (Figure 7a,b). There is no initial decomposition product in the non-C-alloyed specimen (Figure 6a), since no extra reflection spots, other than those from the Cu matrix, are detected by the SAD analysis on the regions (labeled B) beyond α-Fe particles (Figure 7c,d).

The addition of C seems to have no effect on the phases of Fe particles (which will be discussed later), but substantially promotes the appearance of the initial decomposition product. The onset of decomposition of the supersaturated matrix in the C-alloyed specimens is characterized by the appearance of a mottled contrast as seen in Figure 6b–d. SAD analysis indicates the initial decomposition product (labeled C) has an ordered fcc structure with a cube-on-cube OR with the Cu matrix. Superlattice reflections arising from ordering can be seen midway between the {022}_f_ reflections of the product and the transmitted spot, implying solute enrichment on alternate {022}_f_ planes. A similar arrangement of spots along the <022>_f_ direction has been observed in the case of an ordered bcc structure with an N–W OR with the fcc matrix [19]. Therefore, it appears that the initial decomposition product is a precursor to the formation of equilibrium bcc precipitates.

**Figure 8:**
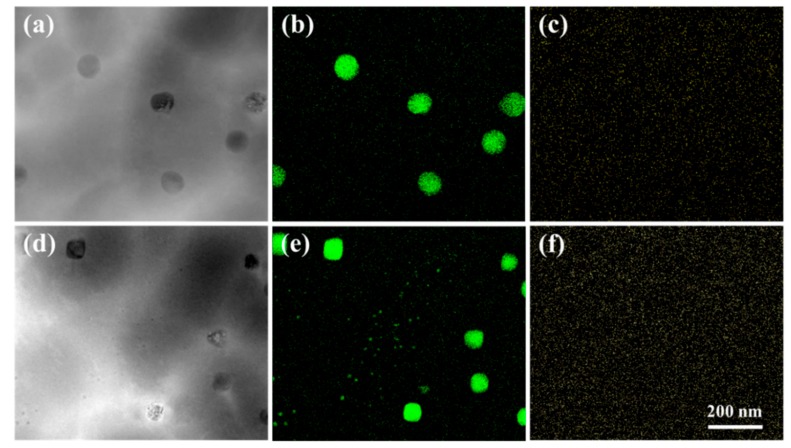
Scanning transmission electron microscopy high-angle annular dark-field (STEM-HADDF) images of (**a**) C00 and (**d**) C01 specimens, respectively, (**b**,**c**) elemental mapping of Fe and C corresponding to (**a**) and (**e**,**f**) elemental mapping of Fe and C corresponding to (**d**).

STEM-HAADF images of the non-C-alloyed and C-alloyed specimens are shown in Figure 8a,d, respectively. The STEM-HAADF image displays the atomic-number-dependent contrast, where the darker area is attributed to the presence of the Fe-rich phase, and the brighter area is the Cu-rich matrix. With the addition of C, the average α-Fe particle size decreases from about 78 to 67 nm. Using EDS mapping in the STEM mode, a large number of nanoscaled Fe-rich particles, resulting from the initial decomposition process, is clearly revealed in the C-alloyed specimens (Figure 8e). The initial decomposition product cannot be found in the non-C-alloyed specimen (Figure 8b), which is consistent with the results of bright-field TEM observations (Figure 6 and Figure 7). The average diameter of the initial decomposition product in the C-alloyed specimen is about 5 nm. No obvious aggregation of the C element is observed in the Fe particles (Figure 8c,f), indicating that no iron carbide is formed during the casting process. As the C addition plays a key role in facilitating the appearance of the initial decomposition product, it is supposed that the C content of the initial decomposition product should be higher than that of the Cu matrix. Unfortunately, due to the low resolution of the EDS mapping analysis, the C content in the initial decomposition product cannot be accurately determined.

## 4. Discussion

**Figure 9:**
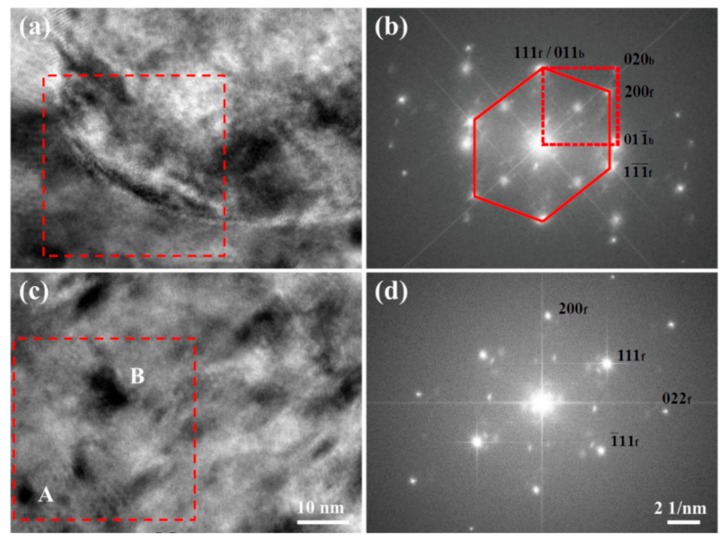
(**a**,**c**) High-resolution transmission electron microscopy (HRTEM) analyses on the Fe particle and initial decomposition product of the C01 specimen, respectively and (**b**,**d**) diffractograms taken from the enclosed area with dotted lines in (**a**) and (**c**), respectively. Zone axis: [$01\bar{1}$]_f_.

The effects of C addition on the second phase particles were discussed here. Figure 9a shows the high-resolution transmission electron microscopy (HRTEM) image of a single Fe particle in the C-alloyed specimen. According to diffractograms obtained by digital Fourier analysis of Figure 9a, it is found that the Fe particle possesses a bcc structure with an N–W OR with the Cu matrix (Figure 9b), which is the same as that observed in the non-C-alloyed specimen (Figure 7a). These results indicate that C addition has no effect on the phases of Fe particles, and both the Fe particles in the non-C-alloyed and C-alloyed specimens are α-Fe particles. However, it is noted that Jeong et al. [13] and Kim et al. [14] reported that C can stabilize the metastable fcc γ-Fe structure, and they found that the Fe particles in the C-alloyed Cu–2.5Fe–0.1P specimens are γ-Fe particles which possess an fcc structure instead of a bcc structure.

The reason for the difference between the phases of Fe particles observed in this study, and that reported in the literature, is mainly related to the Fe particle size. The average Fe particle size in this study is about 67 nm, which is much larger than that reported in the literature (about 20 nm) [13,14]. The bigger particles of Fe in this work compared to studies reported in the literature may be related to the different cooling rate. The average cooling rate of the alloy melts in this study is about 100–110 K/s in the liquid state and 1–4 K/s in the solid state. Unfortunately, the casting condition in the literature is not clear. During the initial stage of decomposition of the supersaturated Cu matrix, small Fe precipitates with an fcc structure and a cube-on-cube OR with the fcc matrix firstly nucleate, due to the much lower interfacial energy of coherent interface that reduces the nucleation barrier compared to incoherent or semi-coherent interfaces [20]. This can be confirmed by the TEM observations of the initial decomposition product in the C-alloyed specimens (Figure 6 and Figure 7). However, fcc nuclei are not the stable form of the Fe phase, and they will transform into the stable bcc Fe phase when they are growing. The driving force for this phase transformation is the misfit elastic energy of coherent fcc precipitates, which increases greatly with size while the interface energy becomes less important in the overall energy balance [21]. Generally, the coherent metastable fcc γ-Fe particles can be stable up to 50 nm in size [22,23]. Clearly, the Fe particles in the C-alloyed specimens of this study are too large to maintain their metastable fcc structure, although the stability of fcc γ-Fe is possibly improved by the C addition. Jeong et al. [13] and Kim et al. [14] reported that coherent γ-Fe particles possess a higher strengthening contribution than incoherent α-Fe particles due to the enhanced stress field around γ-Fe particles. However, Guo et al. [15,16] reported that the strengthening contribution of γ-Fe particles is smaller compared with that of α-Fe particles, because the lattice misfit between Cu matrix and γ-Fe is insignificant. How the phase state of Fe particles affects the properties of the alloys still needs further investigation.

Initial decomposition product is observed in the C-alloyed specimens but not found in the non-C-alloyed specimen, indicating C addition accelerates the kinetics of Fe precipitation. The decomposition product in its various stages of evolution with different sizes and contrast features was further investigated by the HRTEM observation. As shown in Figure 9c, the relatively smaller entities (labeled A) exhibit a black dot contrast, while the other larger entities (labeled B) have a lobe-lobe contrast. Figure 9d shows the Fourier transform pattern of Figure 9c. The beam orientation in Figure 9c is directly in the [$01\bar{1}$]_f_ direction of the Cu matrix, and the intensity of the (200)_f_ and (022)_f_ spots of the decomposition product looks very weak (Figure 9d), which means that the orientation of the product slightly deviates from that of the Cu matrix. This is because the coherent strain energy can be reduced by the slight rotation of Fe crystals during precipitation in the matrix. A large number of initial decomposition product in various orientations lead to the observed SAD patterns with the overall cube-on-cube OR as shown in Figure 6e. A curious observation here is that the reflections are due to the decomposition product having a unit cell larger than that of the matrix. The changes in the dimensions of the fcc cell are similar to that in Cu–Cr–Zr [24] an-d Cu–Be–Co [25] alloy systems which could be attributed to the ordering of solute atoms. The SAD patterns of the decomposition product are also similar with that from the Cu_2_O phase. However, STEM-HAADF images (Figure 8) indicate that the initial decomposition product is mainly comprised of the Fe element, so that it can be confirmed that the SAD patterns are that from the Fe-rich phase which probably contains some C. In addition, the lattice parameters of the decomposition product are also different from that of Cu_2_O. For example, the measured value of the inter-planer spacing for the {200} planes of the decomposition product is about 0.201 ± 0.002 nm, which is smaller than the value of Cu_2_O (~0.212 nm), according to PDF2-2004 34-1354.

## 5. Summary and Conclusions

Cu–1.8Fe–0.02P alloys with an addition of 0 wt.%, 0.01 wt.%, 0.05 wt.% and 0.1 wt.% C were cast by a process of vacuum induction melting. The C-alloyed specimens have nearly the same C contents (0.011–0.015 wt.%) although different amounts of C (0.01–0.10 wt.%) were added into the alloy melts. C addition can induce columnar-to-equiaxed transition and grain refinement, but seems to have no obvious effect on the Fe_3_P particles. α-Fe particles possessing a bcc structure were observed in both the non-C-alloyed and C-alloyed specimens, which satisfy the N–W OR, (111)_f_//(011)_b_, [$01\bar{1}$]_f_//[100]_b_, to the matrix. C is reported to be an γ-Fe stabilizer in the literature. However, the Fe particles in the C-alloyed specimens of this study are too large (67 nm) to maintain their metastable fcc structure, although the stability of fcc γ-Fe is possibly improved by the C addition. The presence of C tends to encourage the finer distribution of α-Fe particles and facilitate the appearance of the initial decomposition product which has an fcc structure with a cube-on-cube OR with the Cu matrix. The results indicate that C addition accelerates the kinetics of Fe precipitation. 

## Figures and Tables

**Table 1:** The measured compositions of the alloys under investigation (wt.%).

Alloy	Fe	P	C	Cu
Cu–1.8Fe–0.02P (C00)	1.79	0.025	0.001	Balance
Cu–1.8Fe–0.02P–0.01C (C01)	1.79	0.020	0.011	Balance
Cu–1.8Fe–0.02P–0.05C (C05)	1.78	0.056	0.013	Balance
Cu–1.8Fe–0.02P–0.10C (C10)	1.78	0.026	0.015	Balance
